# Acidic Microenvironment Aggravates the Severity of Hepatic Ischemia/Reperfusion Injury by Modulating M1-Polarization Through Regulating PPAR-γ Signal

**DOI:** 10.3389/fimmu.2021.697362

**Published:** 2021-06-21

**Authors:** Wei Ding, Yunfei Duan, Zhen Qu, Jiawei Feng, Rongsheng Zhang, Xiaodong Li, Donglin Sun, Xiaoying Zhang, Yunjie Lu

**Affiliations:** ^1^ Hepatopancreatobiliary Surgery Department, The Third Affiliated Hospital of Soochow University, Changzhou First People’s Hospital, Changzhou, China; ^2^ General Surgery Department, Wujin Hospital Affiliated with Jiangsu University, Changzhou, China; ^3^ Hepatobiliary Surgery Department, Nanjing Eight One Hospital, Nanjing, China

**Keywords:** hepatic ischemia-reperfusion injury, macrophage, polarization, PPAR, acidic microenvironment, STAT6

## Abstract

Hepatic injury induced by ischemia and reperfusion (HIRI) is a major clinical problem after liver resection or transplantation. The polarization of macrophages plays an important role in regulating the severity of hepatic ischemia/reperfusion injury. Recent evidence had indicated that the ischemia induces an acidic microenvironment by causing increased anaerobic glycolysis and accumulation of lactic acid. We hypothesize that the acidic microenvironment might cause the imbalance of intrahepatic immunity which aggravated HIRI. The hepatic ischemia/reperfusion injury model was established to investigate the effect of the acidic microenvironment to liver injury. Liposomes were used to deplete macrophages *in vivo*. Macrophages were cultured under low pH conditions to analyze the polarization of macrophages *in vitro*. Activation of the PPAR-γ signal was determined by Western blot. PPAR-γ agonist GW1929 was administrated to functionally test the role of PPAR-γ in regulating macrophage-mediated effects in the acidic microenvironment during HIRI. We demonstrate that acidic microenvironment aggravated HIRI while NaHCO_3_ reduced liver injury through neutralizing the acid, besides, liposome abolished the protective ability of NaHCO_3_ through depleting the macrophages. In vivo and vitro experiment showed that acidic microenvironment markedly promoted M1 polarization but inhibited M2 polarization of macrophage. Furthermore, the mechanistic study proved that the PPAR-γ signal was suppressed during the polarization of macrophages under pH = 6.5 culture media. The addition of PPAR-γ agonist GW1929 inhibited M1 polarization under acidic environment and reduced HIRI. Our results indicate that acidic microenvironment is a key regulator in HIRI which promoted M1 polarization of macrophages through regulating PPAR-γ. Conversely, PPAR-γ activation reduced liver injury, which provides a novel therapeutic concept to prevent HIRI.

## Introduction

Hepatic ischemia/reperfusion injury (HIRI) is the main cause of hepatic insufficiency and failure after liver surgery (1, 2), HIRI causes a series of liver abnormalities, from liver insufficiency to liver nonfunction (3). As reported, the expression of immune-related cytokines was abnormal when acute organ injury was caused by HIRI (4, 5).

The key role in the pathogenesis of HIRI is mainly the inflammation caused by the innate immune response of Kupffer cells (KC) (6). KCs, a type of macrophages, are resident in the liver, which can serve as outposts of hepatic homeostasis (7). In the course of aseptic hepatitis, activated KCs secrete inflammatory cytokines and chemokines and attract other inflammatory cells into the damaged tissue, resulting in increased inflammation (8). The pro-inflammatory M1 type and anti-inflammatory M2 type are the different functional states of macrophages. STAT1 and IRF5 control the M1 macrophage phenotype, whereas M2 macrophage polarization is regulated by STAT6, IRF4, and PPAR-γ (9).

Under ischemia conditions, the promotion of anaerobic glycolysis and accumulation of lactic acid may cause the generation of acidic microenvironment while the pH values of blood or tissues go down to the PH of 6.0 to 6.5. A recent study showed that such a change in the microenvironment may suppress the generation of T regulatory cells (Tregs) and cause an immune imbalance which aggravated HIRI (10). Therefore, the acidic microenvironment is one of most important factors leading to HIRI. However, the relationship between acidic microenvironment and macrophages in HIRI has not yet been investigated. So, the present study performed a series of experiments to determine the function of acidic microenvironment in regulating macrophages in the pathogenesis of HIRI.

## Materials and Methods

### Animals

We placed 8-week-old male mice (C57B/6J) under SPF conditions. These mice were treated and sacrificed at the specified time point according to the protocol approved by the Institutional Animal Care and Use Committee of Soochow University.

### Hepatic Ischemia/Reperfusion Injury Model

Mice were as the previous study indicated (11). The specific construction method was as follows. After anesthesia with 3.5% chloral hydrate (0.1 ml/10 g), a midline incision was made on the abdomen. Non-invasive vascular clamp was used to clamp the left and middle branches of portal vein and hepatic artery for 60 min, and the liver was 70% ischemia. Reperfusion was then started by removing the clamp. The sham group goes through the same procedure in addition to using vascular clamps. For some experiments, macrophages were depleted by injecting clodronate liposomes through tail vein (200 μl, Amsterdam, The Netherlands) 24 h before the model was established. At the indicated time, mice liver tissue and blood were collected.

### KCs Isolation and Cell Culture

Mice were anesthetized, and livers were perfused with perfusion buffer (HBSS) *in situ via* the portal vein, followed by 0.27% collagenase IV (Sigma, Saint Louis, MO, USA). Perfused livers were dissected, and single-cell suspensions were generated through a 70-μm cell filter. The suspension was added with 40 ml of DMEM supplemented with 10% FBS, 10 mM HEPES, 2 mM GlutaMax, 100 U/ml penicillin, and 100 mg/ml streptomycin and standing for 15 min at 37 °C. Then, the adherent cells were left for further experiments while the non-adherent cells were removed. After 6 h of cultured, the KCs or the samples of supernatants were collected for further analysis.

### Immunohistochemical Staining

Liver macrophages were identified with primary rat anti-mouse F4/80 and CD11b, and neutrophils were identified with Ly6G mAb (BD Biosciences, San Jose, CA, USA). Then, the cells were incubated with biotinylated anti-goat IgG, followed by immunoperoxidase (ABC Kit, Vector), according to the specification. The numbers of positive-stained cells were counted blindly in 10 high-power field (HPF) sections.

### Immunofluorescence Staining

LC3B, iNOS, and CD206 in KCs were identified by indirect immunofluorescence staining using rabbit anti-mouse LC3B mAb, anti-mouse iNOS mAb, and anti-mouse CD206 mAb (Cell Signaling Technology, MA, USA). After washing, the KCs which were premounted with VECTASHIELD medium with DAPI (Vector) were incubated with secondary antibody goat anti-mouse Texas Red-conjugated IgG (Sigma, Saint Louis, MO, USA). The numbers of positive-stained cells were counted blindly in 10 high-power field (HPF) sections.

### Quantitative RT-PCR and Western Blot Analysis

RNA and proteins were acquired following the manufacturer’s instructions. Then the PCR and WB assays were run by the standard protocol. Primers are shown as below:

iNOS, 5′- TCTTTCCGTCCGAAGACAGAT -3′
and 5′- TTGTAGTCGTAAGACCTCGATGA -3′;
TNF-α, 5′- TCTTCTCATTCCTGCTTGTGG-3′
and 5′- GGTCTGGGCCATAGAACTGA-3′;
IL-6, 5′- GGCGGATCGGATGTTGTGAT-3′
and 5′- GGACCCCAGACAATCGGTTG-3′;
Arg-1, 5′- CTGAGAGATTCAAGGCAAGAGG-3′
and 5′- GAACGCGCTATCTTACCCCAG-3′;
Mrc2, 5′- ATCCAGGGAAACTCACACGGA-3′
and 5′- GCGCTCATCTTTGCCGTAGT-3′;
IL-10, 5′-GTTACTTGGGTTGCCAAG-3′
and 5′-TTGATCATCATGTATGCTTC-3′;
Gapdh, 5′-CCATCTTCCAGGAGCGAGATC-3′
and 5′-GCCTTCTCCATGGTGGTGAA′.

### Statistical Analysis

Stata software (version 11.0) was utilized to perform the analysis. Data were expressed as mean ± standard deviation (SD), the differences between groups were analyzed by either the paired t-test or ANOVA test (both one-way ANOVA test and two-way ANOVA test). In addition, we use Bonferroni or LSD as a *post hoc* test. *P* values less than 0.05 (two-tailed) was considered statistically significant.

## Results

### HIRI Induced Acidification and Dysfunction of the Liver Microenvironment

**Figure 1:**
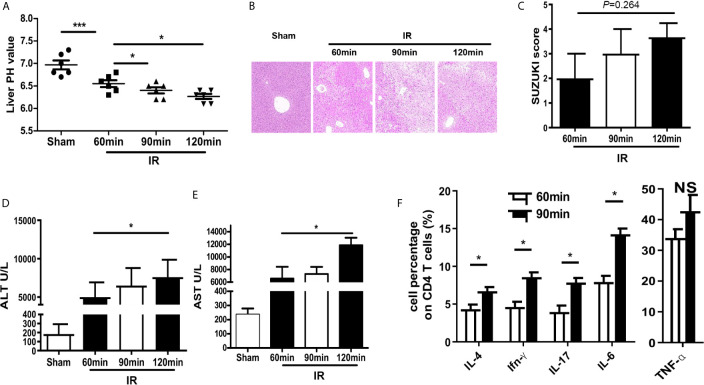
HIRI induced acidification and dysfunction of liver microenvironment. **(A)** pH value of injured liver lobe by time point after reperfusion in mice (one-way ANOVA with Bonferroni as a *post hoc* test, n=6). **(B)** Representative images of liver injury in mice [hematoxylin and eosin (HE) staining, 200×] by light microscopy. **(C)** Suzuki scores for liver tissues in each group (one-way ANOVA with Bonferroni as a *post hoc* test, n=3). Levels of ALT **(D)** and AST **(E)** in sham and IR groups (one-way ANOVA with Bonferroni as a *post hoc* test, n=4 for **D**, n=3 for **E**). **(F)** Cytokine expression on CD4+ T cells derived from blood was tested through flow cytometry (paired t test, n=4). *p < 0.05; ***p < 0.001.

A mouse model of partial hepatic ischemia/reperfusion with different ischemia time interval was established. After reperfusion, injured liver lobe and blood specimens were collected at 60, 90, and 120 min. HE staining of liver tissue and detected ALT and AST in the serum were performed to assess the degree of liver injury. Meanwhile, the pH value of the ischemic liver lobe was also measured. Compared with the sham group, the PH value of injured liver was significantly decreased in the IR group; besides, pH was getting lower accompanied with the increase of ischemia time interval (**Figure 1A**). The pathological results of HE staining showed that the Suzuki scores of mouse livers were increased over time, although the analysis of ANVOA showed no significance (*P*=0.264) in the IR group (**Figures 1B, C**). The values of ALT and AST were upregulated with the increase of ischemia time interval in the IR group (**Figures 1D, E**). Cytokine expression plays an important role to the pathogenesis of HIRI (12–14), so we evaluated the expression of the cytokine on CD4 T cells, data showed that compared with the 60-min ischemia model, 90 min ischemia/reperfusion model presented higher inflammatory cytokine expression such as IL-4, IFN-γ, IL-17, and IL-6 (**Figure 1F**). These results demonstrate that HIRI induced acidification and dysfunction of the liver microenvironment.

### Sodium Bicarbonate Injection Reverses HIRI Through Regulating Acidic Microenvironment by Moderating Macrophages

**Figure 2:**
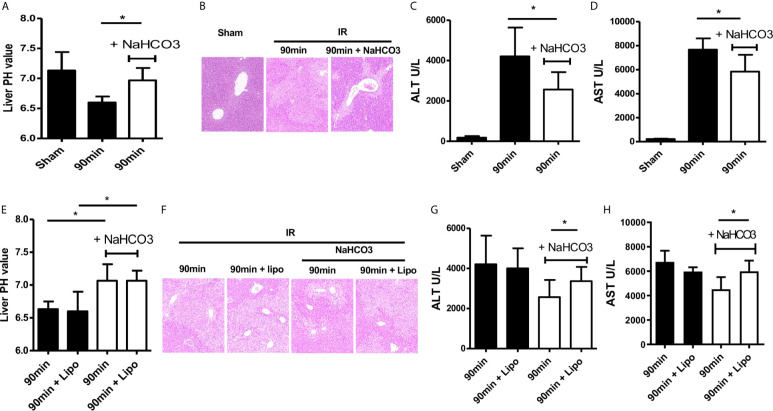
Sodium bicarbonate injection reverse HIRI through regulating acidic microenvironment by moderating macrophages. Mice were injected with sodium bicarbonate the same time while the HIRI model were established. **(A)** pH value of injured liver lobe at each time point after reperfusion in mice (one-way ANOVA with Bonferroni as a *post hoc* test, n=3). **(B)** Representative images of liver injury in mice [hematoxylin and eosin (HE) staining, 200×] by light microscopy. Levels of ALT **(C)** and AST **(D)** in sham and IR groups (one-way ANOVA with Bonferroni as a *post hoc* test, n=3). Liposome was injected through tail vein 24 h before the HIRI model was established to abolish the effect of macrophages. Mice were injected with sodium bicarbonate the same time while the HIRI model were established. **(E)** pH value of injured liver lobe at each time point after reperfusion in mice (two-way ANOVA with Bonferroni as a *post hoc* test, n=3). **(F)** Representative images of liver injury in mice [hematoxylin and eosin (HE) staining, 200×] by light microscopy. Levels of ALT **(G)** and AST **(H)** in sham and IR groups (two-way ANOVA with LSD as a *post hoc* test, n=3). *p < 0.05.

Administration of sodium bicarbonate to increase the intra-tumoral pH had been evaluated as an effective anti-acidic therapeutic strategy (15). Therefore, we tested the protective ability of sodium bicarbonate on regulating intrahepatic pH under conditions of HIRI. Sodium bicarbonate (100 μl/10 g, pH 8.2) was injected through the tail vein at the beginning of ischemia. NaHCO_3_ injection recovered the decrease of intrahepatic pH comparing with the IR group (**Figure 2A**). HE staining of liver tissue showed that NaHCO_3_ injection reduced the degree of injury comparing with the IR group (**Figure 2B**). Consistent with this result, the values of ALT and AST were decreased with the injection of NaHCO_3_ which indicated that NaHCO_3_ reduced liver injury through anti-acidic therapy (**Figures 2C, D**).

Next, we analyzed the role of macrophages during acidic microenvironment-mediated HIRI. In order to assess whether NaHCO_3_ exerts a protective effect of HIRI depending on controlling KCs, we used the liposome to treat KCs (16). Liposome was injected 24 h before the model was established. Then, we detected pH of the ischemic liver tissues, stained liver tissue by HE, and collected serum ALT and AST values. There is no difference in pH value with or without liposome injection (**Figure 2E**), HE staining showed that liposome treatment abolished the protective effect of NaHCO_3_ in HIRI (**Figure 2F**). The ALT and AST change also proved that while NaHCO_3_ reduced the injury of the liver, liposome injection aggravated the injury (**Figures 2G, H**). Taken together, our data indicate that Sodium bicarbonate injection reverses HIRI through regulating acidic microenvironment by modulating macrophages.

### Sodium Bicarbonate Reduces Innate Immune and KC Polarization in HIRI

**Figure 3:**
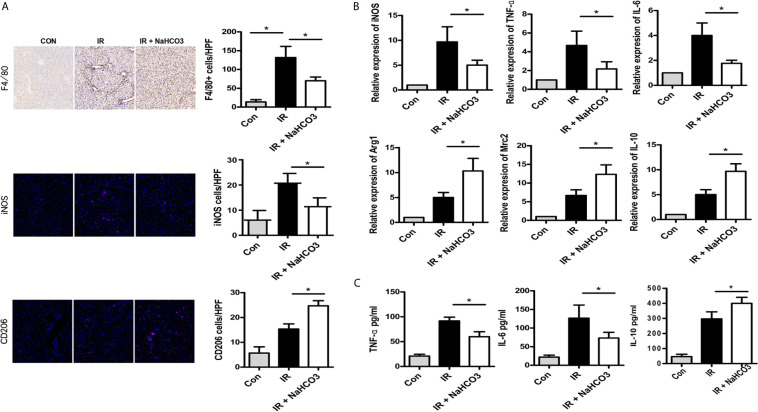
Sodium bicarbonate reduce innate immune and KC polarization in HIRI. Mice were injected with sodium bicarbonate the same time while the HIRI model were established. **(A)** The infiltration of macrophages and M1/M2 cells was analyzed by immunohistochemical and immunofluorescence staining (one-way ANOVA with Bonferroni as a *post hoc* test, n=3). **(B)** The expressions of inflammatory genes in liver tissues were measured by quantitative RT-PCR (one-way ANOVA with Bonferroni as a *post hoc* test, n=3). **(C)** The levels of inflammatory cytokines in serum were measured by ELISA (one-way ANOVA with Bonferroni as a *post hoc* test, n=3). *p<0.05.

We further assessed the effects of sodium bicarbonate on regulating macrophage infiltration by immunohistochemical and immunofluorescence staining. Intrahepatic macrophages (F4/80) were increased in the IR group and reduced in the NaHCO_3_ group. According to the function of macrophages, macrophages could be categorized into M1 (classical) and M2 (alternative) subtypes broadly (17). Thus, we concluded that the sodium bicarbonate has effect on regulating KCs M1/M2 polarization. iNOS and CD206 were the key markers for M1 and M2 separately, so we detected the phenotype of macrophages through immunofluorescence staining, which showed that while in the IR group, more macrophages presented M1 like macrophages, sodium bicarbonate moderated the macrophages to polarize to M2 like macrophages (**Figure 3A**). To confirm the result, we derived intra-macrophages from each group, RT-PCR was used to evaluate the expression of M1 and M2 markers. In the IR model, iNOS, TNF-α, and IL-6 were upregulated in the liver while sodium bicarbonate decreased the transcription of above mRNAs (**Figure 3B**). In contrast, Arg-1, Mrc2, and IL-10 were increased in the NaHCO_3_ group comparing with the IR group. At the same time, we observed that serum cytokine expression for a different group. The results showed that TNF-α and IL-6 were lower expressed, while IL-10 was higher expressed in the NaHCO_3_ group (**Figure 3C**). Therefore, sodium bicarbonate reduces innate immune function by reducing pro-inflammatory TNF-α, IL-6 levels, and KC polarization in HIRI.

### Acidic Microenvironment Regulates M1/M2 Polarization in Response to LPS Condition by PPAR-γ

**Figure 4:**
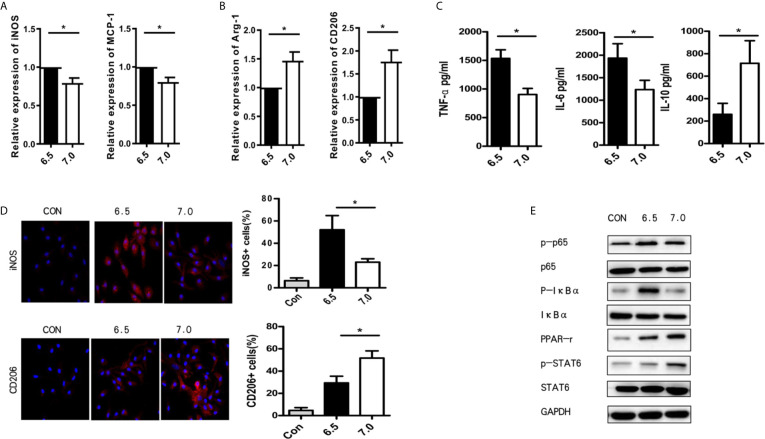
Acidic microenvironment regulates M1/M2 polarization in response to LPS condition by PPAR-γ. Macrophages were isolated from B6 mice. M1 markers (*iNOS and MCP-1*) **(A)** and M2 markers (*Arg-1* and *CD206*) **(B)** of gene induction were measured by quantitative RT-PCR after 6 h of acidic or normal condition treatment (paired t test, n=3). **(C)** Isolated macrophages from different experimental groups were cultured for 24 h. Then, the levels of TNF-α, IL-6, and IL-10 protein in the culture supernatant were detected using ELISA (paired t test, n=3). **(D)** Representative immunofluorescence staining images of iNOS and CD206 in macrophages (one-way ANOVA with Bonferroni as a *post hoc* test, n=3). **(E)** The expression of NF-kB and PPAR-γ signaling in different groups as determined by western blot analysis. *p < 0.05.

In order to further understand the role of acidic microenvironment in regulating macrophages, we plated and cultured macrophages, which isolated from B6 mice, *in vitro* without LPS. As the 90-min ischemia/reperfusion injury model may cause a decrease of intra-hepatic pH to 6.5, we use the culture media with a pH value of 6.5 to mimic the acidic microenvironment *in vitro*. Indeed, after 6 h culture meant, macrophages stimulated with LPS in the acidic group (pH = 6.5) showed a higher expression of M1 markers (*iNOS* and *MCP-1*) but a lower expression of M2 markers (*Arg-1* and *CD206*) in comparison to the normal (pH = 7.0) group. However, Arg-1 and CD206 were increased, while iNOS and MCP-1 were decreased when sodium bicarbonate was added (**Figures 4A, B**). Then, TNF-α, IL-6, and IL-10 protein expression levels were analyzed during macrophage stimulation through ELISA. As shown in **Figure 4C**, the expression of TNF-α and IL-6 was enhanced in the acidic group, while the expression of IL-10 was reduced in the normal group. We also verified the polarization of macrophages by immunofluorescence staining. The results showed that the acidic group promoted the iNOS^+^ M1 cells and suppressed CD206^+^ M2 cells. It demonstrated that acidic microenvironments preferentially polarized macrophages into the M1-like phenotype (**Figure 4D**). Increasing evidence suggests that the polarization of macrophages can be regulated through the NF-Kb and PPAR-γ pathway (18, 19). Subsequently, we verified whether the acidic microenvironment participates in polarization through regulating PPAR-γ. Macrophages were pretreated with acidic or normal culture media and afterwards the PPAR-γ related makers were detected using western blot. As shown in **Figure 4E**, we discovered that acid environment observably reduced the activation of PPAR signaling pathway while normal condition enhanced the activation of PPAR-γ and the phosphorylation of STAT6. Besides, acidic conditions promoted the phosphorylation of p-NF-kBp65 and p-IkBa (**Figure 4**) than normal group. These results suggest that acidic microenvironment regulates M1/M2 polarization in response to LPS condition by PPAR-γ.

### PPAR-γ Agonist GW1929 Suppressed M1 Polarization Induced by the Acidic Microenvironment *In Vitro*

**Figure 5:**
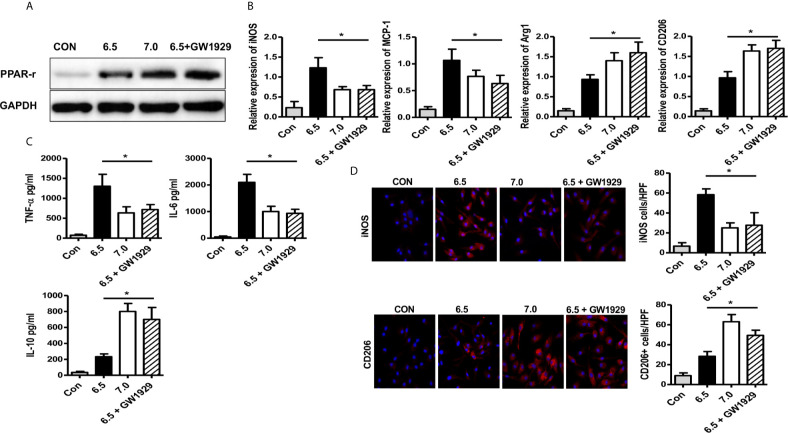
PPAR-γ agonist GW1929 suppressed M1 polarization induced by acidic microenvironment *in vitro*. Macrophages were isolated from B6 mice and GW1929 was added to enhance the expression of PPAR-γ. M1 markers (*iNOS and MCP-1*) **(A)** and M2 markers (*Arg-1* and *CD206*) **(B)** of gene induction were analyzed by quantitative RT-PCR after 6 h of acidic or normal condition treatment (one-way ANOVA with Bonferroni as a *post hoc* test, n=3). **(C)** Isolated macrophages from different experimental groups were cultured for 24 h, and TNF-α, IL-6, and IL-10 protein were measured in the culture supernatant by ELISA (one-way ANOVA with Bonferroni as a *post hoc* test, n=3). **(D)** Representative immunofluorescence staining images of iNOS and CD206 in macrophages (one-way ANOVA with Bonferroni as a *post hoc* test, n=3). *p < 0.05.

GW1929 was an agonist for PPAR-γ which could improve the expression of PPAR-γ (**Figure 5A**). For *in vitro* experiments, macrophages isolated from B6 mice were cultured without the addition of LPS, and GW1929 (5 nM) was added at the same time. After 6 h of culture meant, GW1929 suppressed the polarization of macrophages to M1 by decreasing the expression of *iNOS and MCP-1*, what is more, increased the expression of *Arg-1* and *CD206* (**Figure 5B**). Cytokine expressed by M1 including TNF-α, IL-6 was also reduced under the treatment of GW1929, while the secreting of IL-10 was upregulated (**Figure 5C**). Immunofluorescence staining indicated that GW1929 inhibited iNOS expression of macrophages and upregulated the level of CD206 during acidic media treatment (**Figure 5D**), which proved that GW1929 suppressed M1 polarization in the acidic microenvironment. We demonstrate that PPAR-γ agonist GW1929 suppressed M1 polarization induced by the acidic microenvironment *in vitro*.

### PPAR-γ Agonist GW1929 Suppressed M1 Polarization Induced by Acidic Microenvironment *In Vivo*

**Figure 6:**
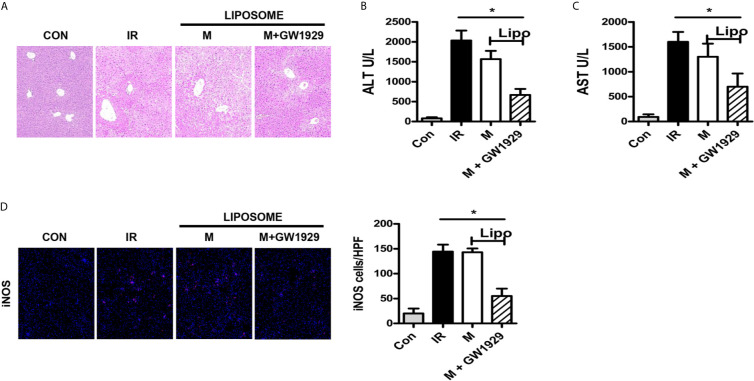
PPAR-γ agonist GW1929 suppressed M1 polarization induced by Acidic microenvironment *in vivo*. **(A)** Representative images of liver injury in mice [hematoxylin and eosin (HE) staining, 200×] by light microscopy. Levels of ALT **(B)** and AST **(C)** in sham and IR groups (one-way ANOVA with Bonferroni as a *post hoc* test, n=3). **(D)** Representative immunofluorescence staining images of iNOS in macrophages (one-way ANOVA with Bonferroni as a *post hoc* test, n=3). *p < 0.05.

Finally, we set up an *in vivo* experiment to analyze the effect of GW1929 in regulating the polarization of macrophages in the HIRI murine model. Clodronate liposomes were injected through tail vein 24 h before the model was established to abolish the effect of macrophages. Macrophages isolated from B6 mice were pretreated with GW1929 to enhance the expression of PPAR-γ, the cells were injected through a tail vein the same day when the HIRI model was built. Data showed that group injected with GW1929 treated macrophages presented strong protective ability compared with IR group or macrophage alone injected group including the HE staining for the liver lobe and ALT, AST level in the serum for each group (**Figures 6A–C**). Additionally, enhanced iNOS levels were detected through immunofluorescence staining, which showed that GW1929 treated macrophages presented a strong anti-acidic effect by suppressing the polarization of intrahepatic macrophages (**Figure 6D**). To conclude, PPAR-γ agonist GW1929 suppressed M1 polarization induced by Acidic microenvironment *in vivo*.

## Discussion

Liver diseases, especially tumors or cirrhosis, were widely used the liver resection and liver transplantation to treat in recent years. HIRI most commonly occurs during liver resection or transplantation and is still the major factor contributing to postsurgical liver dysfunction and liver failure. HIRI severely limits the use of marginal liver donors and the development of extensive hepatectomy. The mechanism of HIRI has been widely studied, but the mechanism was still unclear. The factors/pathways, including anaerobic glycolysis, oxidative stress, intracellular calcium overload, liver KCs, and neutrophil activation, and secretion of cytokines and chemokines, were involved in this process.

For a long time, people have recognized the vital physiological role of acid-base balance in maintaining normal cellular homeostasis (20). As the extracellular pH is lowered, numerous cellular reactions would decrease, including cytoplasmic- and membrane-related enzyme activities, ion transport activity, protein and DNA synthesis, and cAMP and calcium levels (21). Previous researches have always focused on the effect of acid-base environment on lymphocytes in tumors. It is interesting to find that extracellular acidosis can both stimulate and suppress the activity of immune cells. For instance, while the lower PH promotes the expression of nitric oxide synthase in macrophages (22) and the activity of neutrophils (23), it also inhibits cytotoxic natural killer cells (24). Recently, Gan proved that acidic microenvironment decreased Tregs and increased the severity of HIRI (11). Therefore, the extracellular acidic microenvironment may have different effects on macrophages.

KC is the resident macrophages of the liver, accounting approximately for 10-15% of all liver cells (25). KCs locate in the liver sinusoids and engage in various liver diseases and injuries as the first-line defense against invading bacteria and virus. Many studies have reported the role of KCs M1/M2 polarization in various liver injuries (26). During HIRI, HO-1 suppressed M1 polarization and protects liver injury (27); Maspin regulated M1 polarization through activation of the NF-κB signal (28); besides, ATF6, ATF3 was also involved in HIRI and macrophage polarization (29, 30).

The results of the present study showed that HIRI induced liver acidification which aggravated the liver injury, NaHCO_3_ protected HIRI through reverse the acidic microenvironment while liposome abolished the protective ability of NaHCO_3_ through depleting the macrophages. In vivo and vitro experiment showed that acidic microenvironment markedly promoted M1-type polarization and inhibited M2-type polarization of macrophage. Furthermore, the mechanism study proved that the PPAR-γ signal was suppressed during the polarization of macrophages under PH=6.5 culture media. The addition of PPAR-γ agonist GW1929 inhibited M1 polarization under acidic environment and reduced HIRI.

PPAR-γ is a type 2 nuclear receptor located on the human chromosome, encoded by the PPARG gene. Initial studies have shown that this receptor has an important effect on fat cells and insulin, and is one of the research targets for the treatment of diabetes (31, 32). At present, the study of PPAR-γ on HIRI is not yet clear. Liver transplantation studies have shown that HIRI does not induce PPARγ expression in the liver (33). However, in the mouse model of warm ischemia, the PPAR-γ agonist pioglitazone reduces the accumulation of proinflammatory cytokines, chemokines and neutrophils to protect the liver injury. The liver damage was more severe in PPAR-γ-KO mice generated HIRI models (34). Losartan (35), retinol-binding protein 4 (36) have also been reported to reduce HIRI through the PPARγ pathway. Still, multiple studies have confirmed that PPAR-γ was involved in the regulation of macrophage function: PPARγ activation inhibited the release of TNFα, IL-1, and IL-6 from macrophages (37, 38) and regulated polarization of macrophages to M1 (39).

In conclusion, the present study demonstrates that acidic microenvironment plays an important role in immune balance modulation which directly leads to HIRI injury. PPAR-γ signal participated in the regulation effect of acidic microenvironment on macrophages. Our findings provided ideas for further confirming the pathological relationship between HIRI and acidic microenvironment and suggested that acidic microenvironment should be considered as a potent target for HIRI.

## Data Availability Statement

The data sets presented in this study can be found in online repositories. The names of the repository/repositories and accession number(s) can be found in the article/supplementary material.

## Ethics Statement

The animal study was reviewed and approved by the institutional animal care and use committee of Soochow University.

## Author Contributions

Conception and design: WD, YD, and ZQ. Analysis and interpretation: WD and RZ. Data collection: WD and JF. Writing the article: WD, YD, ZQ, DS, XZ, and YL. Critical revision of the article: WD, YD, ZQ, DS, XZ, and YL. Final approval of the article: WD, YD, ZQ, DS, XZ, and YL. Statistical analysis: WD, YL, and XL. Obtained funding: WD and YL. Overall responsibility: DS. All authors contributed to the article and approved the submitted version.

## Funding

This work was supported by the National Natural Science Foundation of China (81971504), Post-Doctoral Special Foundation of China (2020M670065ZX), Post-Doctoral Foundation of Jiangsu Province (2020Z021), Scientific and Technological Projects for Young Talents, Changzhou Health and Family Planning Commission (QN201809), Young Talent Development Plan of Changzhou Health Commission (2020- 233), Young Talent Development Plan of Changzhou Health Commission (CZQM2020118), the Development Foundation of Affiliated Hospital of Xuzhou Medical University (XYFY2020016), DAAD-K. C. WONG Funding (57501535), Changzhou Social Development Funding (CE20205038), and Lindau Follow-up Funding (GZ1600).

## Conflict of Interest

The authors declare that the research was conducted in the absence of any commercial or financial relationships that could be construed as a potential conflict of interest.
